# High malaria transmission sustained by *Anopheles gambiae* s.l. occurring both indoors and outdoors in the city of Yaoundé, Cameroon

**DOI:** 10.12688/wellcomeopenres.14963.1

**Published:** 2018-12-23

**Authors:** Patricia Doumbe-Belisse, Carmene Sandra Ngadjeu, Nadege Sonhafouo-Chiana, Abdou Talipouo, Landre Djamouko-Djonkam, Edmond Kopya, Roland Bamou, Jean Claude Toto, Souleyman Mounchili, Raymond Tabue, Parfait Awono-Ambene, Charles Sinclair Wondji, Flobert Njiokou, Christophe Antonio-Nkondjio

**Affiliations:** 1 Malaria Research Laboratory, OCEAC, Yaoundé, Cameroon; 2 University of Yaoundé 1, Yaoundé, Cameroon; 3 University of Buea, Buea, Cameroon; 4 University of Dschang, Dschang, Cameroon; 5 National Malaria Control Programme, Yaoundé, Cameroon; 6 Liverpool School of Tropical Medicine, Liverpool, L3 5QA, UK

**Keywords:** Malaria, urbanization, Anopheles, transmission, Yaoundé, Cameroon

## Abstract

**Background: **Malaria remains a major public health problem in Cameroon; however, despite reports on the adaptation of anopheline species to urban habitats, there is still not enough information on malaria transmission pattern in urban settings. In the frame of a larval control trial in the city of Yaoundé, we conducted baseline surveys to assess malaria transmission dynamics in this city.

**Methods: **Adult mosquitoes were collected indoors and outdoors using CDC light traps and human landing catches from March 2017 to March 2018 in 30 districts of Yaoundé, Cameroon. Mosquitoes were sorted by genus and identified to the species level using PCR. The TaqMan method and ELISA were used to determine mosquito infection status to *Plasmodium*. Bioassays were conducted to assess female *Anopheles gambiae* susceptibility to insecticides.

**Results: **A total of 218,991 mosquitoes were collected. The main malaria vectors were An. *gambiae* s.l. (n=6154) and *An. funestus* s.l. (n=229). Of the 1476 *An. gambiae* s.l. processed by PCR, 92.19% were *An. coluzzii *and 7.81% *An. gambiae*. *An. funestus* s.l. was composed of 93.01% (173/186) *An. funestus* and 4.84% (13/186) *An. leesoni*. The average biting rate of anopheline was significantly high outdoor than indoor (P=0.013). Seasonal variation in mosquito abundance and biting rate was recorded. The infection rate by *Plasmodium falciparum* was 2.13% (104/4893 mosquitoes processed). The annual entomological inoculation rate was found to vary from 0 to 92 infective bites/man/year (ib/m/y). Malaria transmission risk was high outdoor (66.65 ib/m/y) compared to indoor (31.14 ib/m/y). *An. gambiae* s.l. was found highly resistant to DDT, permethrin and deltamethrin. High prevalence of the West Africa *kdr* allele 1014F was recorded and this was not found to influence *An. gambiae* s.l. infection status.

**Conclusion**: The study suggests high malaria transmission occurring in the city of Yaoundé and call for immediate actions to improve control strategies.

## Introduction

The world population has registered unprecedented growth during the last decades with Africa and Asia displaying the most important rates ^1^. The fast demographic growth registered so far has increased the path of urbanization across Africa. There are more and more cities with over a million inhabitants and megacities with 5 to 10 million inhabitants ^2^. Because in most parts of Africa the rapid development of cities is often associated with unplanned urbanization, this could have important public health implications for vector-borne diseases such as malaria ^1, 3^. Urban malaria is now considered as an emerging health problem in Africa and is receiving further consideration in many countries ^4– 7^. In Cameroon the population is estimated at 25 million inhabitants and over ¼ of this population is considered to live in the two main cities of the country, Yaoundé and Douala ^8^. Yaoundé, the capital city of Cameroon has seen its population multiplied by 6 over the two last decades and now has a population estimated at about 3 million inhabitants ^8^. The city is situated at the heart of the equatorial forest region and irrigated by numerous rivers. In the 1950s, indoor spraying campaigns conducted in Yaoundé and it surroundings, resulted in a decrease transmission level and Plasmodium prevalence in the population to near to zero ^9^. At 30 years after interruption of these campaigns, very limited transmission level was still recorded in the city centre. In the 1990s, in the districts of Obili and Essos, Manga et *al.* ^10^ reported no infected mosquitos, whereas moderate level of transmission varying from 14 to 30 infected bites per man per year was recorded in the districts of Nkolbikok and Nkolbisson ^11^. Similar transmission levels (3–33 infected bites per man per year) were recorded few years later in the central district of Dakar ^12, 13^. Contrasting pattern was, however, recorded at the city periphery or nearby rural settings where transmission levels varying from 277 to 350 infected bites per man per year were recorded ^14– 16^.

Bionomic studies conducted from 2000 onwards indicated important changes on vector populations. In the cities of Douala and Yaoundé, increased distribution of mosquito in polluted habitats and artificial breeding sites was reported ^17^. Moreover, mosquitoes breeding in polluted areas or cultivated sites were found to be more resistant to insecticides compared to those breeding in unpolluted sites ^17, 18^. Rapid evolution of insecticide resistance was also recorded in vector populations and an increase in the prevalence of resistance genes such as *kdr* was observed ^19, 20^. The exposition of mosquito to xenobiotics was found to shape the distribution of *Anopheles gambiae* and *Anopheles coluzzii* in the cities of Douala and Yaoundé with *Anopheles coluzzii* more tolerant to pollutants being predominant in the urban centre whereas *Anopheles gambiae* less tolerant to pollutants being prevalent in rural settings ^21, 22^. Laboratory experiments provided evidence supporting a possible influence of this adaptation on the vectorial competence of these two species, with *An*. *coluzzii* being more competent to transmit malaria parasite compare to *An. gambiae* ^23^.

In addition to the rapid demographic growth of the city, important physical changes have occurred during the last decade with the construction of new roads and buildings, creation of parks and the drainage of rivers. Yet it is still unknown whether the current adaptation of malaria vectors to the urban environment and physical changes occurring in the city are affecting malaria transmission pattern and vector biting behavior. The present study was conducted in 30 districts of the city of Yaoundé to assess the trend of malaria transmission and to capture spatial and temporal variations.

## Methods

### Study site

**Figure 1.**
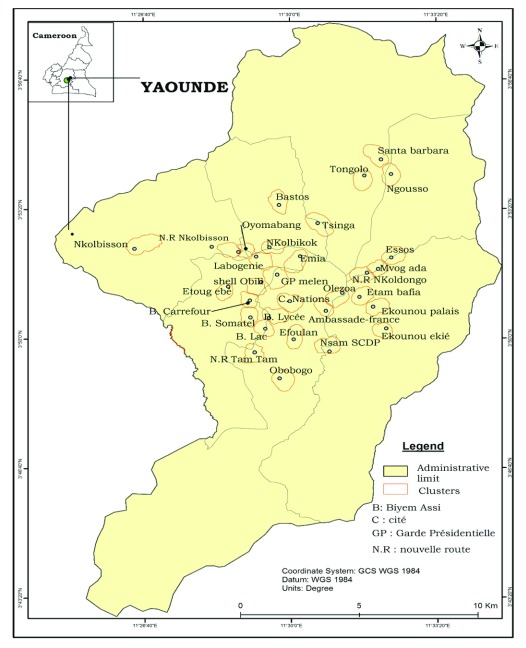
Map of Yaoundé city showing study sites. Source: National Institute of Cartography, Cameroon.

The study was conducted in Yaoundé, the capital city of Cameroon (3° 52' 12 N; 11° 31' 12 E). Yaoundé is situated 726 meters above sea level and receives over 1700 mm of rainfall annually. Yaoundé features an equatorial climate with two raining seasons extending from March to June and from September to November lasting 7 to 8 months. Although Yaoundé is situated in the equatorial forest domain, the extension of settlements has significantly reduced the forest cover which is now restricted to the nearby rural area. The city extends on 20 km wide and about 25 km long. Yaoundé landscape comprises highland and lowland areas, which are irrigated by several rivers. Lowland areas are exploited during the dry season for agriculture. Main rivers present in the city are rivers Mfoundi, Ekozoa, Biyeme and Mefou, Adult mosquitoes’ collections were performed in thirty districts ( Figure 1).

### Mosquitoes sampling

Adult mosquitoes were sampled using Human Landing Catches (HLC) and the Centres for Disease Control and Prevention Light Traps (CDC-LTs) both indoors and outdoors. Collections were performed once every 2 months from March 2017 to March 2018.

There were four HLC volunteers trained to collect mosquitoes landing on their legs. Collections were performed from 19:00 to 06:00 AM indoors and outdoors. During each night two teams of collectors were used to avoid bias due to sleep and tiredness.

A total of 16 to 20 CDC- LTs were placed indoors and outdoors in 10 to 15 houses per district. Collections were undertaken from 19:00 to 06:00 during 3 consecutive days per district per month.

The study was conducted under the ethical clearance No. 2018/06/1039/CE/CNERSH/SP delivered by the Cameroon National Ethics (CNE) Committee for Research on Human Health Ref N°D30-172/L/MINSANTE/SG/DROS/TMC of 4 April 2017. All volunteers participating to human landing catches signed a written inform consent form indicating their willingness to take part to the study and received free malaria prophylaxis.

### Mosquito processing

Once collected, anophelines were separated from culicines using the morphological identification keys of Edwards *et al.* ^24^. Anopheline species were identified using morphological identification keys of Gillies and De Meillon ^25^ and Gillies and Coetzee ^26^. Mosquitoes belonging to the *Anopheles gambiae* complex were further processed by PCR ^27^ to identify between *Anopheles coluzzii* and *Anopheles gambiae*, the two members of the complex found in Yaoundé. Molecular identification of members of the *Anopheles funestus* was conducted using the protocol of Koekemoer *et al.* ^28^. DNA extracted from the wings and legs according to the Livak method ^29^ was used for the analysis. Each anopheline specimen was stored individually in a numbered Eppendorf tube containing dessicant, archived and kept in a freezer at −20°C. The heads and thoraxes of female anophelines were tested for the presence of circumsporozoite protein (CSP) of *Plasmodium falciparum* by ELISA, as described by Wirtz *et al*. ^30^ or using the Taqman method ^31^.

### Insecticide susceptibility tests

Susceptibility of *An. gambiae* s.l. to 0.75% permethrin, 0.05% deltamethrin, 0.1% bendiocarb and 4% DDT was assessed using the WHO guidelines ^32^ at temperatures of 25±2°C and 70–80% relative humidity. Insecticide susceptibility tests were performed with 2- to 4-day-old unfed females. Batches of 20 to 25 mosquitoes per tube were exposed to impregnated papers for 1 hour. The number of mosquitoes knocked down by the insecticide was recorded every 10 minutes during exposure. After exposure, mosquitoes were fed with a 10% glucose solution and the number of dead mosquitoes was recorded 24 hours post-exposure. Tests using untreated papers were conducted as well (controls). The mortality rates were corrected using the Abbot formula ^33^ whenever the mortality rate of the controls was between 5 and 20%. Susceptibility and resistance levels were assessed according to the World Health Organization criteria ^32^. Mosquito were classified into these three groups: insecticide resistant (<90%), possible insecticide resistant (90–97%) and insecticide susceptible (>98%).

To detect the presence of the *kdr* alleles (L1014F and L1014S) conferring resistance to DDT and pyrethroids, DNA extracted from a sub sample of *An. gambiae* s.l. females was screened using the TaqMan assay ^34^.

### Data analysis

Mosquito densities were compared between seasons, collection sites and districts. Biting rate (number of bites per person per night, b/p/n) was calculated as the number of mosquitoes caught in one night divided by the number of collectors. The infection rate was calculated as the number of infected mosquitoes divided by the total number of *Anopheles* processed. The entomological inoculation rate (EIR) (the number of infected bites per person per night, ib/p/n) was calculated by multiplying the infection rate by the biting rate/night. To assess linear correlations between the two collection methods, the Pearson correlation coefficient was used to calculate the average number of mosquitoes collected nightly by the CDC-LT and HLC methods. Prior to analysis, the average number for each catch (x) was transformed to Y = log(x+1). To compare methods and determine if mosquito abundance was affected by the sampling efficiency of each method used, the ratio of the number of mosquitoes in LT to the number of mosquitoes in HLC [Log(HLC+1)-Log(LT+1)] was plotted against the average abundance [Log(HLC+1)+Log(LT+1)]/2 as described by Overgaard *et al.* ^35^.

The knock down time for 50% of mosquitoes (Kd _50_) and 95% of mosquitoes (Kd _95_) representing the time when 50% or 95% of the mosquitoes exposed are knocked down, was calculated using the WINDL32 version 2.0 software. Chi-square test was used to compare proportions, student tests and one way ANOVA were used to compare averages. All these tests were performed using SPSS software (SPSS version 20.0) and R software version 3.4.0. The level of significance of each test was set at α < 0.05. The coefficient r of Pearson was used to assess correlation between CDC-LT and HLC.

## Results

All raw data associated with this study are available on OSF ^36^.

### Mosquito collection

***Mosquito diversity and abundance.*** Among the 218,991 specimens identified morphologically, *Culex* spp. was the most abundant (96.48%; n=211,993) followed by *An. gambiae* s.l. (2.81%; n=6154) and *Mansonia* spp. (0.47%; n=1026). *An. funestus* s.l. was less abundant (0.10%; n=229) ( Table 1). Overall, 187, 404 (85.58%) mosquitoes were collected using a total of 9968 CDC-LTs placed per night per house and 31,587 (14.42%) were collected using a total of 348 human-night collectors. The average density of mosquitoes collected using CDC-LTs, was high indoors than outdoors (t = 8.28, d=29.6, P< 0.0001) while the trend was not significantly different between outdoor and indoor catches using HLC (t = -0.68, df =58, P=0.2508).

**Table 1.** Mosquitoes sampled in Yaoundé using CDC-LTs and HLC from March 2017 to March 2018.

Species	CDC-LT				HLC				Total	
	Indoor		Outdoor		Indoor		Outdoor			
	N	%	N	%	N	%	N	%	N	%
*An. funestus*	199	0.13	15	0.05	5	0.30	10	0.06	229	0.10
*An.gambiae* s.l.	3,542	2.29	414	1.26	772	5.08	1,426	8.71	6,154	2.81
*An. ziemanni*	4	0.00	8	0.02	0	0.00	0	0.00	12	0.01
**Total anopheline**	** 3,745**	**2.42**	**437**	**1.33**	**777**	**5.11**	**1,436**	**8.77**	**6,395**	**2.92**
*Aedes* spp.	182	0.12	52	0.16	14	0.09	15	0.09	263	0.12
*Culex* spp *.*	150,020	97.09	32,054	97.46	14,381	94.56	14,838	90.59	211,293	96.48
*Coquillettidia*	6	0.00	8	0.02	0	0	0	0.00	14	0.01
*Mansonia* spp.	563	0.36	337	1.02	36	0.24	90	0.55	1026	0.47
**Total culicines**	**150,771**	**97.58**	**32,451**	**98.67**	**14,431**	**94.89**	**14,943**	**91.23**	**212,596**	**97.08**
**Overall**	**154,516**	**70.56**	**32,888**	**15.02**	**15,208**	**6.94**	**16,379**	**7.48**	**218,991**	**100.00**

**Figure 2.**
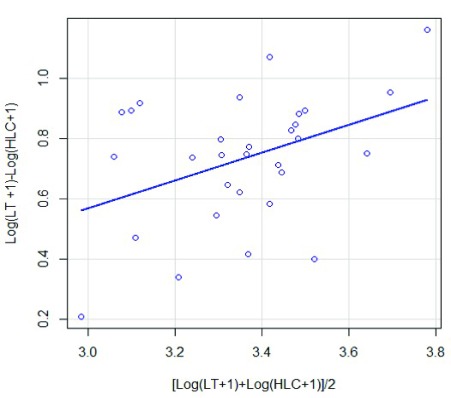
Relationship between CDC-LT catches, human landing catches (HLC) and mosquito abundance.

Correlation analysis between CDC-LT and HLC catches using the Pearson correlation coefficient, indicated that when all mosquitoes were considered, CDC-LTs catches were positively and significantly correlated with those of HLC (r = 0.408, P = 0.025). When mosquitoes were separated into the two most common genera ( *Culex* and *Anopheles*), the correlation was positive but not significant for *Culex* (r = 0.324, P = 0.081) whereas it was negative for *Anopheles* (r = −0.013, P = 0.94). Comparing the relative sampling efficiencies of the two methods against mosquito abundance ( Figure 2), it appears that there was a significant tendency for increased mosquito abundance with the ratio of LT to HLC (P < 2.2×10 ^-16^).

**Figure 3.**
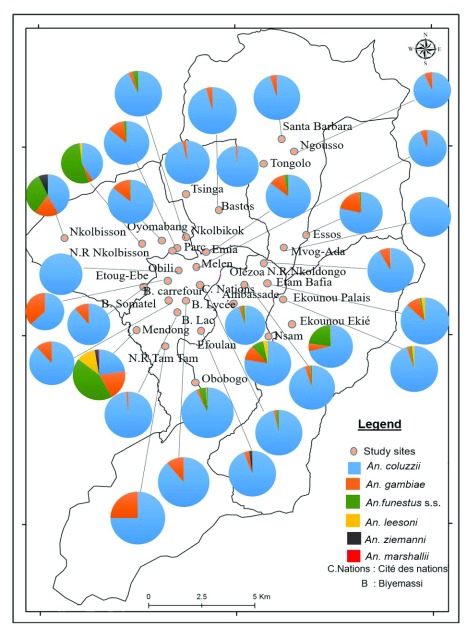
Spatial distribution of *Anopheles* species in the city of Yaoundé. Source: National Institute of Cartography, Cameroon.

***Distribution of Anopheles species.*** Anopheline species recorded includes *An. ziemanni* mainly found in Nkolbisson, *An. gambiae* s.l. and *An. funestus* s.l. Out of 1476 *An. gambiae* s.l. analysed, 92.19 % (n=1351) were *An. coluzzii* and 7.81% (n=125) *An. gambiae*. *An. coluzzii* was highly predominant across the city whereas *An. gambiae* was found in a sizeable density at Nkolbisson, Emia and Etoug ebe ( Figure 3). Amongst the 186 *An. funestus* s.l. tested, 4.84% (n=8) were *An. leesoni* and 93.01% (n= 173) *An. funestus* s.s. The small proportion of *An. leesoni* observed was mainly recorded from Ekounou palais, Etam Bafia and NVR Nkolbisson. *An. funestus* was found in 21 sites out of 30 districts. Significant variation in species densities was recorded between districts (P=0.008).

### Seasonal variation of anopheline species densities collected with CDC-LTs

**Figure 4.**
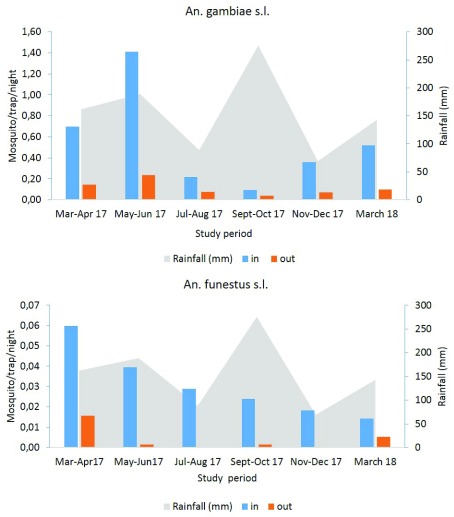
Seasonal variation of *An. gambiae* s.l. and *An. funestus* densities in Yaoundé using CDC-LT.

Seasonal variation of anopheles densities was recorded both indoors and outdoors. Our results indicated high densities indoor than outdoor whatever the species and the sampling period. The indoor density of *An. gambiae* s.l. estimated by CDC-LTs varied from 1.41 mosquitoes/trap/night in May–June period (short raining season) to 0.09 mosquitoes/trap/night in September-October period (long raining season). The same trend was observed with mosquitoes sampled outdoor during the same period with densities varying from 0.23 mosquitoes/trap/night in May-June to 0.04 mosquitoes/trap/night in September-October period ( Figure 4). *An. funestus* densities were found to increase, at the onset of the short rainy season in March–April 17 (0.06 mosquitoes/trap/night (indoor) and 0.02 mosquitoes/trap/night (outdoor)). Low densities were recorded during the short rainy season of March 2018 (0.01 mosquitoes/trap/night both in and outdoor) ( Figure 4).

### Seasonal variation of anopheline species densities collected using HLC

**Figure 5.**
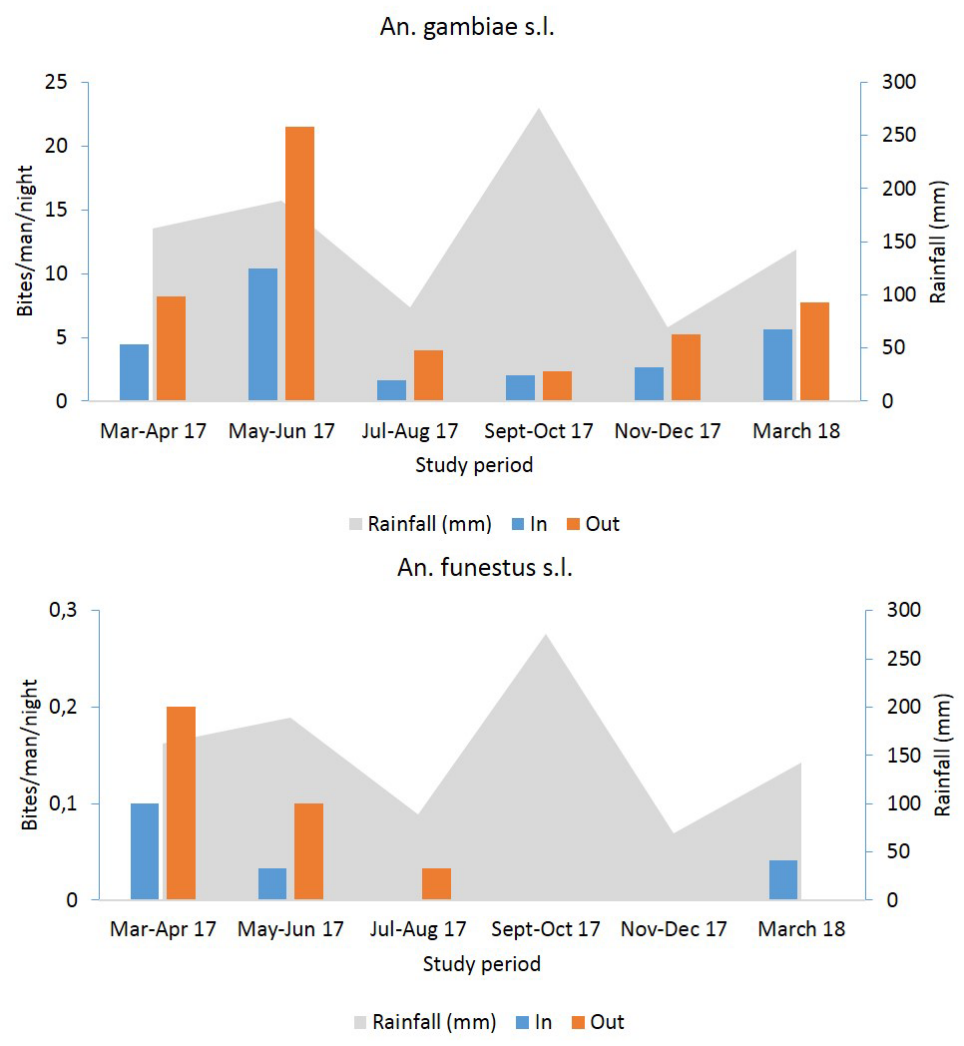
Seasonal variation of *An. gambiae* s.l. and *An. funestus* densities after human landing catches.

High *An. gambiae* s.l. biting densities were recorded outdoor in May-June (21.53 b/m/n) during the short rainy season then decrease to 2.37 b/m/n in September-October at the onset of the long rainy season. Indoor collections declined from 10.43 b/m/n in May-June during the short rainy season to 1.67 b/m/n in July-August period during the short dry season ( Figure 5).

*An. funestus* was more abundant at the onset of the short rainy season with a 0.2 b/m/n outdoors and 0.1 b/m/n indoors, then its outdoor biting rate fell during the short dry season to 0.033 b/m/n while the indoor biting rate was reduced to 0 b/m/n during the same period ( Figure 5).

The average biting rate for *An. gambiae* s.l. varied from 4.44 b/m/n indoor to 8.20 b/m/n outdoor and the difference was significant (P = 0.013). Concerning *An. funestus* its biting rate was 0.03 b/m/n and 0.06 b/m/n for indoor and outdoor respectively; the difference was not significant (P = 0.733).

### Indoor and outdoor biting behaviour of *An. gambiae* and *An. funestus*

**Figure 6.**
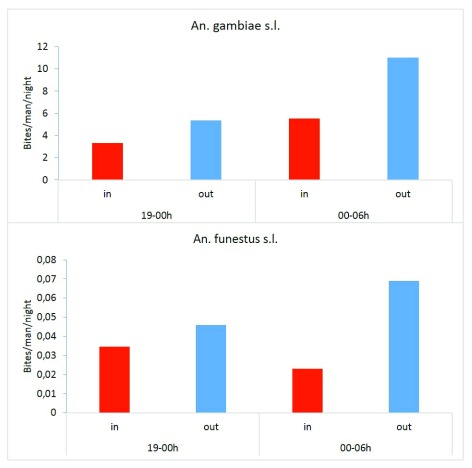
*An. gambiae* and *An. funestus* night biting densities after human landing catches.

*An. gambiae* s.l. densities collected during the second part of the night (24:00-06:00) were high compare to those of the first part of the night both indoors and outdoors ( Figure 6). The difference was not significant for both indoor and outdoor catches (P > 0.26). For *An. funestus,* densities collected outdoor from 24:00 to 06:00 were not significantly different from those of the first part of the night (t=-0.42, df=8.33, P=0.68). Similarly, no significant difference was recorded between indoor and outdoor collections (t=0.47, df= 9.9811, P=0.65).

### Plasmodium infection in mosquitoes

Of the 4893 anopheline (223 *An. funestus* s.l., 4661 *An. gambiae* s.l. and 09 *An. ziemanni*) screened for *Plasmodium falciparum* presence, 104 specimens were positive (103 *An. gambiae* s.l. and 01 *An. funestus* s.l.). Of the 103 *An. gambiae* s.l. detected positive 91% (n=94) were *An. coluzzii* and 9% (n=9) *An. gambiae*. The overall *P. falciparum* infection rate was 2.13%. Out of the infected mosquitoes, 66 were collected using CDC-LTs while the remaining (n=38) was collected using HLC. The circumsporozoite rates between indoor and outdoor collections are presented in Table 2. *An. gambiae* s.l. infection rate was 2.3% indoor and 2% outdoor. The circumsporozoite rate for *An. funestus* was 0.4%. 

**Table 2.** Sporozoite rates for Anopheles mosquitoes from indoor and outdoor collections in Yaoundé.

Species	Indoor			Outdoor			Total		
	Tested	Inf	% (95% CI)	Tested	Inf	% (95% CI)	Tested	Inf	% (95% CI)
*An. gambiae* s.l.	3,278	76	2.3 (2-3)	1,383	27	2 (1.3-3)	4,661	103	2.2 (1.8-2.7)
*An. funestus* s.l.	199	1	0.5 (0.0133)	24	0	0 (0-15)	223	1	0.4 (0.01-2.5)
*An. ziemanni*	3	0	0 (0-123)	6	0	0 (0-61.5)	9	0	0 (0-41)
Total	3,480	77	2.2 (1.7-3)	1,413	27	1.9 (1.3-3)	4,893	104	2.1 (1.7-2.6)

Inf, infected; %, infection rate; 95% CI, 95% confidence interval.

### Relationship between infection and *kdr* allele presence

Analyses were conducted to compare the prevalence of the West Africa *kdr* allele in infected and non-infected mosquitoes. A total of 143 *An. gambiae* s.l. including 73 positive for *Plasmodium* CSP and 70 non infected were randomly selected and genotyped for *kdr*-West (L1014F) mutation. The L1014F mutation was detected in both groups ( Table 3). There was no significant difference between genotypes to *kdr* gene and mosquito infectious status (P=0.30).

**Table 3.** Relationship between *An. gambiae* s.l. infection status to *Plasmodium falciparum* and *kdr* allele frequencies.

Infection status	N	L1014F *kdr* genotypes			F( *kdr*)	P
		RR	RS	SS		
Infected*	73	45	26	2	0.70	
Non-infected*	70	57	13	0	0.91	*0.30
Overall	143	102	39	2	0.79	

RR, homozygote resistant; RS, heterozygote; SS, susceptible; F( *kdr*), frequency of the *kdr* allele.

### Malaria transmission and EIRs

**Figure 7.**
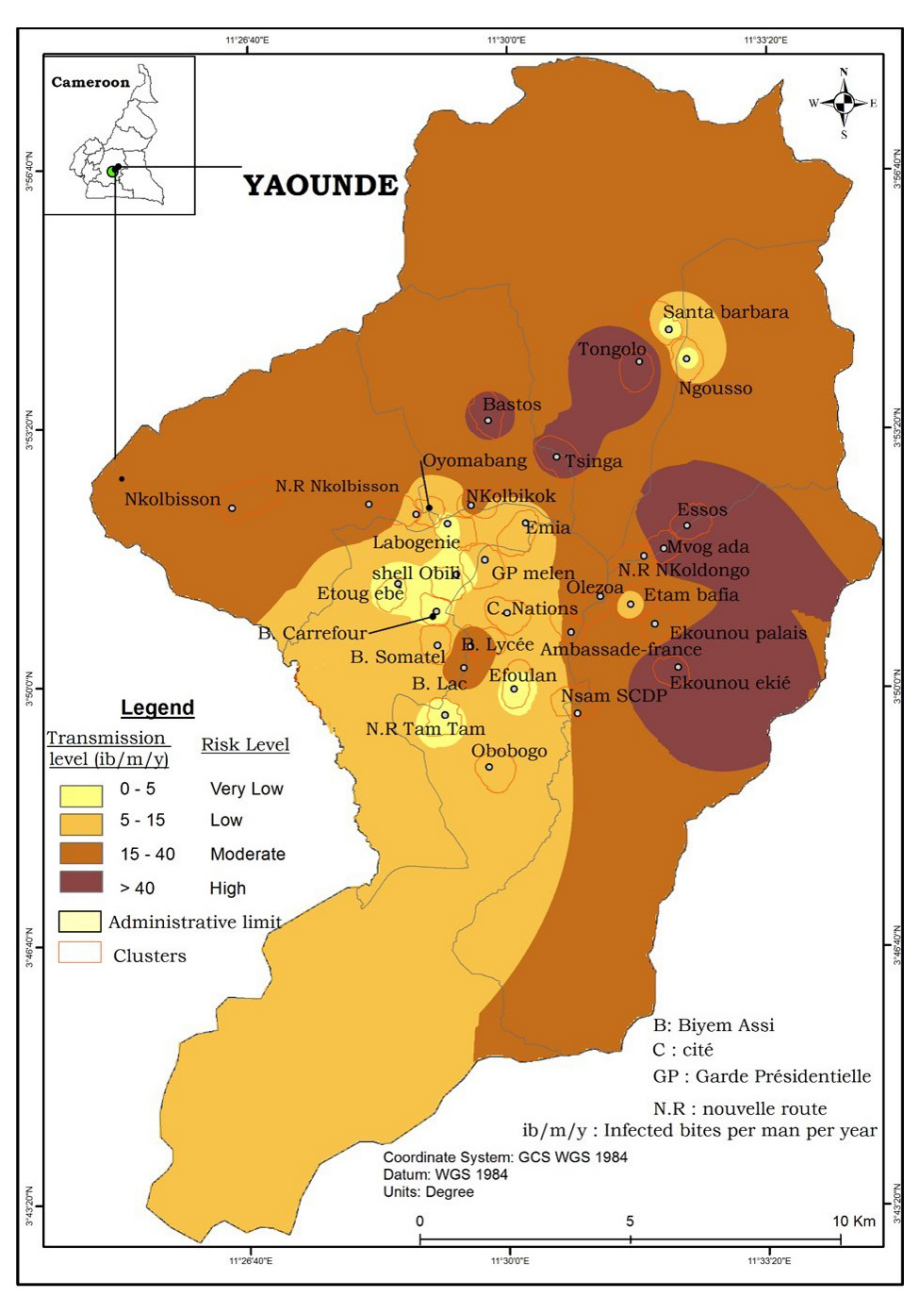
Map showing the risk of malaria transmission in the city of Yaoundé. Source: National Institute of Cartography, Cameroon.

**Figure 8.**
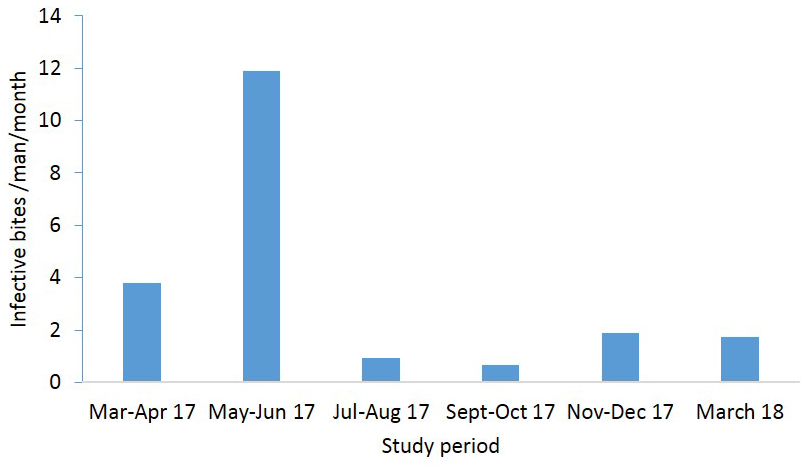
Seasonal variation of the entomological inoculation rate in Yaoundé, from March 2017 to March 2018.

Malaria transmission was found to occur continuously through the city but with different intensities levels according to seasons and districts. The EIR was estimated at 31.14 infected bites/man/year (ib/m/yr) indoor and 66.65 ib/m/yr outdoor. An estimation of malaria transmission risk throughout the city of Yaoundé was conducted and significant differences were recorded between districts ( Figure 7). Very low transmission risk with EIR varying from 0 to 5 ib/m/yr was recorded in nine districts (Tam-tam, Biyemassi Carrefour, Efoulan, Etoug Ebe, Ngousso, Obobogo, Labogenie, Santa Barbara, Obili). Low transmission risk with transmission level varying from 5 to 15 ib/m/yr was recorded in eight districts (Nkolndongo, Ambassade de France, Biyemassi Somatel, Cité des nations, Etam-bafia, Melen, Oyomabang, Emia) while moderate transmission risk (EIR 15 – 40 ib/m/yr) was recorded in seven districts (Biyemassi lycée, Biyemassi lac, Ekounou palais, Nkolbikok, Nkolbisson, NVR Nkolbisson, Oledzoa). High transmission risk with EIR exceeding 40 ib/m/yr was recorded in six districts (Essos, Ekounou Ekié, Bastos, Mvog-Ada, Tongolo, Tsinga) with Ekounou Ekié scoring the highest EIR (92 ib/m/yr). Malaria transmission in the city of Yaoundé was recorded all year long with the intensity varying from one season to the other. The short rainy season of April-June 2017 was the period where most transmission cases were recorded ( Figure 8).

### Insecticide susceptibility bioassay

 Preliminary tests were conducted with *An. gambiae* s.s. Kisumu strain to assess the quality of the impregnated papers. A total of 100 mosquitoes were tested for each insecticide. A mortality rate ranging from 95% for DDT to 100% for permethrin deltamethrin and bendiocarb was recorded. Susceptibility tests were subsequently undertaken with field females raised from larval collection. The mortality rate ranged from 2.85% for DDT to 96.31% (n= 911) for bendiocarb (n = 1709) ( Table 4).

**Table 4.** Susceptibility level to deltamethrin, permethrin, bendiocarb and DDT for *An. gambiae* s.l. populations in Yaoundé.

Insecticides	Kisumu colony		Field populations	
	N	Mortality% (95%CI)	N	Mortality% (95%CI)
Deltamethrin 0.05%	100	100 (81.36-121.63)	2350	23.28 (21.37-25.31)
Permethrin 0.75%	100	100 (81.36-121.63)	486	34.16 (29.16-39.77)
DDT 4%	100	95 (81.36-121.63)	911	2.85 (1.86-4.18)
Bendiocarb 0.1%	100	100 (81.36-121.63)	1709	96.31 (91.72-101.08)

N: number tested, 95%CI : 95% confidence interval

### Genotyping of the West *kdr* mutation

Of the 194 anopheline females (including susceptible, resistant and control) screened for the presence of the West Africa *kdr* allele (L1014F), 187 were detected carrying the allele 39 as homozygotes, 148 as heterozygotes and 7 were homozygote for the wild allele ( Table 5). The frequency of the West Africa *kdr* allele (L1014F) was 0.59. The *kdr* allele frequency was not significantly different between resistant and susceptible samples (χ ^2 ^= 0.039, P= 0.84).

**Table 5.** Frequency of the *kdr* alleles in *An. gambiae* s.l. samples from Yaoundé.

Population	N	L1014F *kdr* genotype			F( *kdr*)	P
		RR	RS	SS		
Survival population*	97	22	70	5	0.59	*0.84
Susceptible specimen*	58	10	46	2	0.57	
Control	39	7	32	0	0.59	
Overall	194	39	148	7	0.59	

N, number tested; RR, homozygote resistant; RS, heterozygote; SS, susceptible; F( *kdr*), frequency of the *kdr 1014F* allele.

## Discussion

High and permanent malaria transmission was recorded in the city of Yaoundé and was consistent with studies conducted so far suggesting urban malaria as a public health threat in most sub-Saharan Africa cities ^1, 6, 37– 39^. The spatial design of the study, covering up to 30 districts, permitted to capture the heterogeneous pattern of malaria transmission risk in the city of Yaoundé varying from very low to high risk zones. This pattern was in accordance with the presence of hotspots areas across the city. According to Robert *et al.* ^40^, malaria transmission in sub-Saharan Africa could be less intense in the city centre with mean annual entomological inoculation rate estimated at 7.1 ib/man/y, whereas it is more intense in the periurban and rural settings, where it could be estimated at 45.8 and 167.7 ib/man/y, respectively. The complexity of malaria situation in Yaoundé could derive from the combination of different factors, including the peculiarity of the city landscape. The city of Yaoundé is composed of an alternation of hills and lowland areas and is irrigated by several permanent rivers. During the rainy season, inundation of floodplains resulting from the overloading of river systems in lowlands areas create suitable habitats for mosquitoes, whereas during the dry season, river channels and/or the exploitation of floodplains for urban agriculture, provide suitable mosquitoes breeding habitats. In addition to these factors the rapid unplanned urbanisation affecting almost all districts characterized by the creation of slums, the absence of drainage system for surface water, the presence of large open space and the development of several activities, such as car washing, urban farming, public and private constructions, create numerous breeding opportunities for mosquito of the *An. gambiae* complex in the urban environment as reported elsewhere ^3, 5, 17, 41– 43^. Similar contrasting pattern of malaria transmission was reported for the city of Libreville with high transmission occurring at the city centre and low transmission at the periphery ^6^. According to the authors, this situation could have resulted from poor housing, poor waste management and slum-like conditions in the urban centre compare to the city periphery. 

Compared to previous studies in Yaoundé which indicated transmission intensity varying between 0 to 33 infected bites/man/yr ^11, 12^, transmission rate reaching up to 92 ib/man/yr was detected in the present study, highlighting the changing pattern of malaria transmission dynamic and vector bionomic in the city of Yaoundé. Indeed, three of the most efficient malaria vectors in Africa *An. gambiae, An. coluzzii* and *An. funestus* were recorded in Yaoundé. However, *An. coluzzii* was by far the most abundant species. Its distribution was in conformity with previous studies conducted so far in Yaoundé suggesting high capacity of adaptation of this species to urban environments and polluted habitats ^17, 18, 21, 22, 44^. *An. funestus* was scarce in the city centre; its presence could be associated with the development of new adaptation capacities or the development of suitable breeding habitats in urban districts. *An. funestus* breed in permanent water collection with emerging vegetation ^45^ and could have benefited from the practice of urban agriculture in swamps to extend its distribution range. The adaptation of *An. funestus* to the urban environment alongside *An. gambiae* and *An. coluzzii* and the changing transmission pattern in Yaoundé all suggest the need for in-depth assessment of main determinants of transmission in order to address sustainably challenges impeding malaria control efforts in this city. 

A high EIR was recorded outdoors, which supports an increased transmission risk outdoors. Similar findings were recorded in several cities across sub-Saharan Africa ^5, 46, 47^. In the case of Yaoundé increased transmission risk outdoor could be related to the development of new activities which keep many people outdoors late. Many commercial activities, schools, leisure and business places are all open during the night and keep people outdoors late. This behaviour could affect the performance of insecticide based interventions used as prevention methods by the population. In Cameroon, long-lasting insecticidal nets (LLINs) are the main measure used for protecting against malaria. It is estimated that over 77% of the population own at least a net and 58% of the population is considered using nets regularly ^48^. Following mass campaigns for distribution of LLINs initiated since 2004, a decrease in the incidence of malaria cases and mortality rates were recorded in Cameroon; however, the rate of decline of the disease incidence has stopped since 2016 ^48^. Similar trend was recorded in the WHO region of the Americas, in South East Asia and Western Pacific and African regions ^49^. The following could point to the limits of current control interventions and the need for new control strategies. Another factor which could be affecting the performance of control interventions is pyrethroid resistance, studies undertaken herein in different districts confirm high resistance of *An. gambiae* populations to deltamethrin, DDT and permethrin. The data was consistent with previous findings and highlights the rapid expansion of insecticide resistance in mosquito population from the city ^17– 19^. In addition to the *kdr* allele, metabolic base mechanisms were reported involved in mosquito resistance to pyrethroid and bendiocarb in Yaoundé ^19, 20, 50, 51^. The presence of resistant alleles was not found to affect the vectorial competency of *An. gambiae* s.l. to *Plasmodium* parasite. The current situation of malaria in Yaoundé requests an adaptation of vector control strategies in light of new challenges. The use of additional control measures such as larval control in an integrated control approach could be indicated in the case of this city where there is a high and focal transmission occurring mainly outdoor, rapid expansion of insecticide resistance and high nuisance due to mosquitoes from the Culex genus.

## Conclusions

The study suggests heterogeneous pattern of malaria transmission in the city of Yaoundé. Several factors including outdoor transmission and increased expansion of insecticide resistance were found to affect control measures the following call for concerted efforts in order to improve malaria control interventions in the city of Yaoundé. The use of a larvicidal strategy, as is planned for the city of Yaoundé, could be well adapted for controlling outdoor and indoor biting mosquitoes and insecticide resistant vectors. As the study indicated there are focal sites in the city more affected than others so increasing control efforts in these sites could be indicated for the sustainable control of malaria in Yaoundé. 

## Data availability

The raw data underlying the findings reported in this study are available on OSF. DOI: https://doi.org/10.17605/OSF.IO/KYXTN ^36^.

Data are available under the terms of the Creative Commons Zero "No rights reserved" data waiver (CC0 1.0 Public domain dedication).
