# Supplementary material for: High malaria transmission sustained by Anopheles gambiae s.l. occurring both indoors and outdoors in the city of Yaoundé, Cameroon
Source: Wellcome Open Res. 2018 Dec 23;3:164. [Version 1] doi: 10.12688/wellcomeopenres.14963.1 (PMC6364383; doi:10.12688/wellcomeopenres.14963.1)
Supplement: Peer review history

# Peer review: Referee response for version 1

**DOI:** 10.21956/wellcomeopenres.16320.r34736

**Published:** 2019-02-04

**Authors:** Joseph Chabi

**Affiliations:** 1 Abt Assosiates /PMI Vectorlink, Massachusetts, MA, USA

The manuscript “ **High malaria transmission sustained by *Anopheles gambiae* s.l. occurring both indoors and outdoors in the city of Yaoundé, Cameroon**” presents the finding of malaria transmission and vector susceptibility in the city of Yaounde, the capital of Cameroon. The study was well designed and the methods well followed to enabling good data collection. However, the language need a small perfection to enable flowed reading as corrected below. Additional details need to be added on the description of the methods.

**Abstract**

 L2: remove “however” and just start the sentence by “Despite”

 L3: remove” not enough information “by “limited information”

**Method**

 Anopheles gambiae susceptibility to insecticides (list the insecticides tested and concentrations)

**Results**

 The average biting rate was “higher”, not “high”.

 Malaria transmission rate was “higher”, not “high”.

 Last sentence of the results “High prevalence of kdr……..infection status” Sentence a bit unclear. What is the link the authors wanted to show between the kdr allele and the prevalence of infection? Sentence to be reformulate to elucidate the idea.

**Introduction**

 Sentence “Because in most parts of Africa………….such as malaria” Remove because and start the sentence by “In most parts of Africa ……which could have …………such as malaria”.

 The population is estimated to, not “at”.

 Yaoundé the capital has seen its population……….., just say “the population of Yaoundé, the capital has been multiplied by six over the two last decades.”

 “Decrease of the plasmodium to near to zero”, consider giving the prevalence unit (decrease of plasmodium prevalence to almost zero malaria/1000/year for example).

 Remove ‘At” 30 years and just write “Thirty years after those campaigns….”

**Methods**

**Mosquito sampling**

- Describe a bit more the collection methods, how many nights for the HLCs, how were the collectors rotated, were the collection houses fixed or changed per collection periods.
- How were the CDC-LT set?, baited or not, distance they were hung, ….

Harmonize the times: 7:00PM to 6:00 AM instead of 19:00 and 6 AM

**Susceptibility test**

 No need to specify that the KD was read every 10mins. This is no more in the new guidelines, so just refer to KD after 60 mins.

 Resistance status: <90% is confirmed resistance, not insecticide resistance, possible or suspected resistance and susceptible for the 2 others. Remove “insecticide”.

**Results**

 Write in *italic* the anopheles in the figure titles.

 The figures may be more understandable if the monthly collection (HBR) can be a curve within the rainfall figure. The bar charts ae not showing the real tendencies. So all the biting cycle figures could be changed from bar charts to curves.

 Also, it has not been mentioned anywhere in the methods that the rainfall incidence was collected throughout the study period. So the author should include how the rainfall data was collected. Was that done per the team during the specific collection period or it’s just general?

 It can be seen that the densities were low when the rainfall peaks, which is a bit contradictory from several published articles. So the authors need to clarify that a bit.

 Concerning An. funestus, “its” biting rate was, just write “the biting rate was”.

**Malaria transmission and EIR**

 Write in full Entomological Inoculation Rate for the first time it used.

 Write continuously “within the city” instead of “through the city”.

 Kindly harmonize the HBR unit (b/m/n) and EIR (ib/m/y).

**Susceptibility test**

 The number of mosquitoes tested in bracket is not well displayed. (DDT 2.85% and 911 tested, note 96.31%).

**Genotyping**

 It should have been interesting to check also the Kdr-East (1014S). Check and see if your DNA are still available for screening if possible.

**Discussion**

 EIR rate estimated to , not “at” 7 ib/m/y (be consistent. Either ib/m/y or ib/man/y).

 Is this an interval or specific values (45.8 and 167.7 ib/m/y)?

 During the rainy season………..created suitable breeding sites for mosquitoes = contradictory with your data as stipulated above.

 However, An. coluzzii was **by far** the most abundant species “remove “by far”.

 The adaptation of An. funestus…………………Yaoundé all suggest….(remove “all”).

 The authors emphasized the LLIN distribution and the 77% coverage in the country. Check and see if you can have the specific percentages for the study site (Yaoundé). We know that the coverage in cities and capital are not similar to the rural and even the net usage. So to highlight well the trend of increasing or change of transmission patterns in the city of Yaoundé, those LLIN data could support your statement.

 Rephrase the sentence: “In addition of the kdr, metabolic resistance mechanisms involved in the resistance………were reported”.

I have read this submission. I believe that I have an appropriate level of expertise to confirm that it is of an acceptable scientific standard.

# Peer review: Referee response for version 1

**DOI:** 10.21956/wellcomeopenres.16320.r34495

**Published:** 2019-01-31

**Authors:** Luc Salako Djogbenou

**Affiliations:** 1 Regional Institute of Public Health, University of Abomey-Calavi, Cotonou, Benin

This article presents the situation of malaria transmission dynamics in space and time from March 2017 to March 2018 in 30 districts of Yaoundé, Cameroon.  This study has shown that the pattern of malaria transmission in the city of Yaoundé is complex and calls for the development of sustainable control strategies.

**Materiel & Methods**

 In the mosquitoes sampling section, it is not clear whether the collection frequency is once every 2 month per district or monthly. The authors need to make this clear in the section.

**Data analysis**

 In this section, please add sentence indicating the calculation of species densities

 collected with CDC-LTs.

**Results**

 Malaria transmission and EIRs

 Please add in this section the criteria (references) which allow the categorization of the transmission level.

I have read this submission. I believe that I have an appropriate level of expertise to confirm that it is of an acceptable scientific standard.
